# GAGA: A New Algorithm for Genomic Inference of Geographic Ancestry Reveals Fine Level Population Substructure in Europeans

**DOI:** 10.1371/journal.pcbi.1003480

**Published:** 2014-02-20

**Authors:** Oscar Lao, Fan Liu, Andreas Wollstein, Manfred Kayser

**Affiliations:** 1 Department of Forensic Molecular Biology, Erasmus MC University Medical Center Rotterdam, Rotterdam, The Netherlands; 2 Department of Medical Statistics and Bioinformatics, Leiden University Medical Center, Leiden, The Netherlands; UCLA, United States of America

## Abstract

Attempts to detect genetic population substructure in humans are troubled by the fact that the vast majority of the total amount of observed genetic variation is present within populations rather than between populations. Here we introduce a new algorithm for transforming a genetic distance matrix that reduces the within-population variation considerably. Extensive computer simulations revealed that the transformed matrix captured the genetic population differentiation better than the original one which was based on the T1 statistic. In an empirical genomic data set comprising 2,457 individuals from 23 different European subpopulations, the proportion of individuals that were determined as a genetic neighbour to another individual from the same sampling location increased from 25% with the original matrix to 52% with the transformed matrix. Similarly, the percentage of genetic variation explained between populations by means of Analysis of Molecular Variance (AMOVA) increased from 1.62% to 7.98%. Furthermore, the first two dimensions of a classical multidimensional scaling (MDS) using the transformed matrix explained 15% of the variance, compared to 0.7% obtained with the original matrix. Application of MDS with Mclust, SPA with Mclust, and GemTools algorithms to the same dataset also showed that the transformed matrix gave a better association of the genetic clusters with the sampling locations, and particularly so when it was used in the AMOVA framework with a genetic algorithm. Overall, the new matrix transformation introduced here substantially reduces the within population genetic differentiation, and can be broadly applied to methods such as AMOVA to enhance their sensitivity to reveal population substructure. We herewith provide a publically available (http://www.erasmusmc.nl/fmb/resources/GAGA) model-free method for improved genetic population substructure detection that can be applied to human as well as any other species data in future studies relevant to evolutionary biology, behavioural ecology, medicine, and forensics.

## Author Summary

Understanding genetic population substructure is important in evolutionary biology, behavioral ecology, medical genetics and forensic genetics, among others. Several algorithms have recently been developed for investigating genetic population substructure. However, detecting genetic population substructure can be cumbersome in humans since most of the genetic diversity present in that species exists among individuals from the same population rather than between populations. We developed a Genetic Algorithm for Genetic Ancestry (GAGA) to overcome current limitations in reliably detecting population substructure from genetic and genomic data in humans, which can also be applied to any other species. The method was validated by means of extensive demographic simulations. When applied to a real, human genome-wide SNP microarray dataset covering a reasonable proportion of the European continent, we identified previously undetected fine-scale genetic population substructure. Overall, our study thus not only introduces a new method for investigating genetic population substructure in humans and other species, but also highlights that fine population substructure can be detected among European humans.

This is a *PLOS Computational Biology* Methods article.

## Introduction

At what degree genetically homogeneous groups of human individuals exist is a long-standing and yet unsolved debate in the scientific community [1]. Answering this question is important for better understanding recent human evolutionary history [1], for reducing the amount of false positives in gene mapping studies [2] and other medical issues [3], and for inferring the bio-geographic origin of unknown persons in forensic investigations [4]. In general, for any species, detecting genetically homogeneous groups can be of relevance in answering questions in evolutionary biology and behavioural ecology. Previously developed methods for estimating average genomic ancestry and detecting genetic population substructure can be broadly classified into two types: model-based ancestry estimation and algorithmic ancestry estimation [5]. The former type aims to estimate the contribution of hypothetically existing ancestral populations to the genome of each specimen tested; popular implementation methods include STRUCTURE [6], ADMIXTURE [5], and FRAPPE [7]. The latter type uses hypothesis-free multivariate techniques, such as Principal Component Analysis (PCA; [8]), classical multidimensional scaling (MDS), or principal coordinates analysis [9], to position each specimen tested in a reduced Euclidean space [10], so that the proximity between specimens can be interpreted as genetic affinity [8]. The coordinates proposed by algorithmic ancestry methods tend to correlate with the geographic sampling location of the tested individuals when applied to human genetic data [11]. Recently, a method called SPA [12] was proposed; it exploits the geographic dependency between allelic frequencies and space to infer the coordinates in a 2D/3D space of a given set of individuals.

However, detecting genetic population substructure can be complex depending on the evolutionary history of the species in question, and certainly in the case of humans. Certain processes such as isolation by geographic distance [13], local genetic adaptation to environmental factors [14], and other factors including cultural ones [15], all impact on the amount of genetic differences observable between individuals within and between populations [16]. In particular, the recent origin of the human species and the even more recent dispersal out of the African continent [17] played a major role in shaping the neutral variation of the human genome with dramatic consequences for the detection of genetic population substructure. Due to our single recent origin, the vast majority (∼85%) of the total genetic differences is explained by variation between individuals within populations [1]. Moreover, the genetic differences between populations usually follow clinal geographic patterns [18], which typically are in agreement with major past migration routes [19], rather than showing sharp discontinuities. For instance, within the European continent, the genetic differentiation between European subpopulations (with exceptions such as European Romani, [20]) is small [21] compared to that found among worldwide populations, and even smaller when sampling within specific sub-regions of Europe [22]. Furthermore, long identical-by-descendent (IBD) genomic tracks that are shared between geographically distant European individuals have been found, suggesting a recent common ancestry of European populations [23]. Finally, individuals from one population tend to have their best genetic-matching partner (as defined by the Best Overall Match (BOM)) far away from their sampling population [24]. Nevertheless, a remarkable correlation between genetic and geographic distance as well as a clinal distribution of genetic diversity on the continental [21], [25] and sub-regional level (i.e., [22]) have been observed within Europe.

Overall, the fact that the vast majority of human genetic variation exists among individuals within populations [21] limits the capacity of existing methods to resolve genetic population substructure at a fine geographic scale and asks for the development of alternative methods for detecting population substructure and genetic ancestry in humans that can also be applied to other species. Recently, a new algorithm implemented in the fineSTRUCTURE software [26] analyzes the shared haplo-blocks between previously phased pairs of individuals. However, genome phasing can be computational intensive [27], especially when a large number of individuals and markers is used. Moreover, despite the current state-of-art of phasing algorithms [27], errors are unavoidable, especially when considering variants at low frequency [28]; furthermore, some prior population information is usually desired [27]. Finally, genomic SNP density is only considerable in the case of humans (and not for all the geographic regions [29])), whereas in other species, such as cattle, a relatively limited number of markers have been described thus far [30].

In the present study, we propose a new matrix distance transformation with the aim to reduce the within-population variation. We conducted extensive computer simulations under two demographic models to test if this aim is achieved. We additionally implemented a genetic algorithm which, in combination with AMOVA statistics, allows searching for the optimal genetic clustering configuration of specimens and populations. We practically test the performance of this new, model-free approach using a genome-wide dataset comprising 2,457 individuals from 23 geographically dispersed subpopulations of Europe. We make this new method for improved genetic population substructure detection publically available as software package for free use.

## Materials and Methods

### Quantifying the amount of genetic differentiation between populations

Our algorithm starts with a genetic distance matrix D computed for each possible pair among N individuals, which in this study is derived from the T1 statistic [31]. The T1 statistic has been shown to be informative for detecting hidden genetic relatedness [32], independently of the (unknown) allelic frequencies in each population [31]. T1 is defined for a given pair of individuals i and j as:
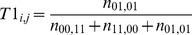
(1)


where n_xx,yy_ denotes the number of SNPs of a particular genotype pattern (i.e. n_00,11_ refers to SNPs where the first individual is homozygous for one allele (0) and the second individual is homozygous for the alternative allele (1)). Under Hardy-Weinberg equilibrium (HWE), the expectancy E(T1)  = 2/3 if both individuals are unrelated from the same population, E(T1) < 2/3 if the individuals are from different populations and E(T1) > 2/3 if they are more related than by chance. We define the distance matrix as D = 1-T1. That is, we set d_i,j_ = 1-T1_i,j_ in order to obtain a genetic distance between individuals i and j.

Individuals can then be classified into populations, and the genetic differentiation between populations quantified using this individual distance by applying the Analysis of Molecular Variance (AMOVA [33]) framework. In analogy to the Analysis Of Variance (ANOVA), the AMOVA framework decomposes the total sum of squares (SS(T)) from the individual distance matrix in sum of squares among populations (SS(AP)) and sum of squares within populations (SS(WP)), so that:
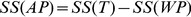
(2)

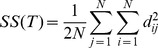
(3)

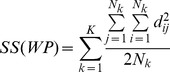
(4)


where K is the number of groups.

The estimated values of population differentiation can be transformed into Fst-like statistics, and reflect demographic parameters such as migration rate or time of population split, among others [34]. However, it has been suggested that within population variance can be as high as the total variance for highly polymorphic markers, resulting in very low values of SS(AP) even if the compared populations have no alleles in common [35]. Meirmans [35] proposed a standardized version of the AMOVA under different scenarios. However, several other genetic dissimilarity statistics can also be used for estimating genetic relatedness between diploid individuals [36], [37].

**Figure 1:**
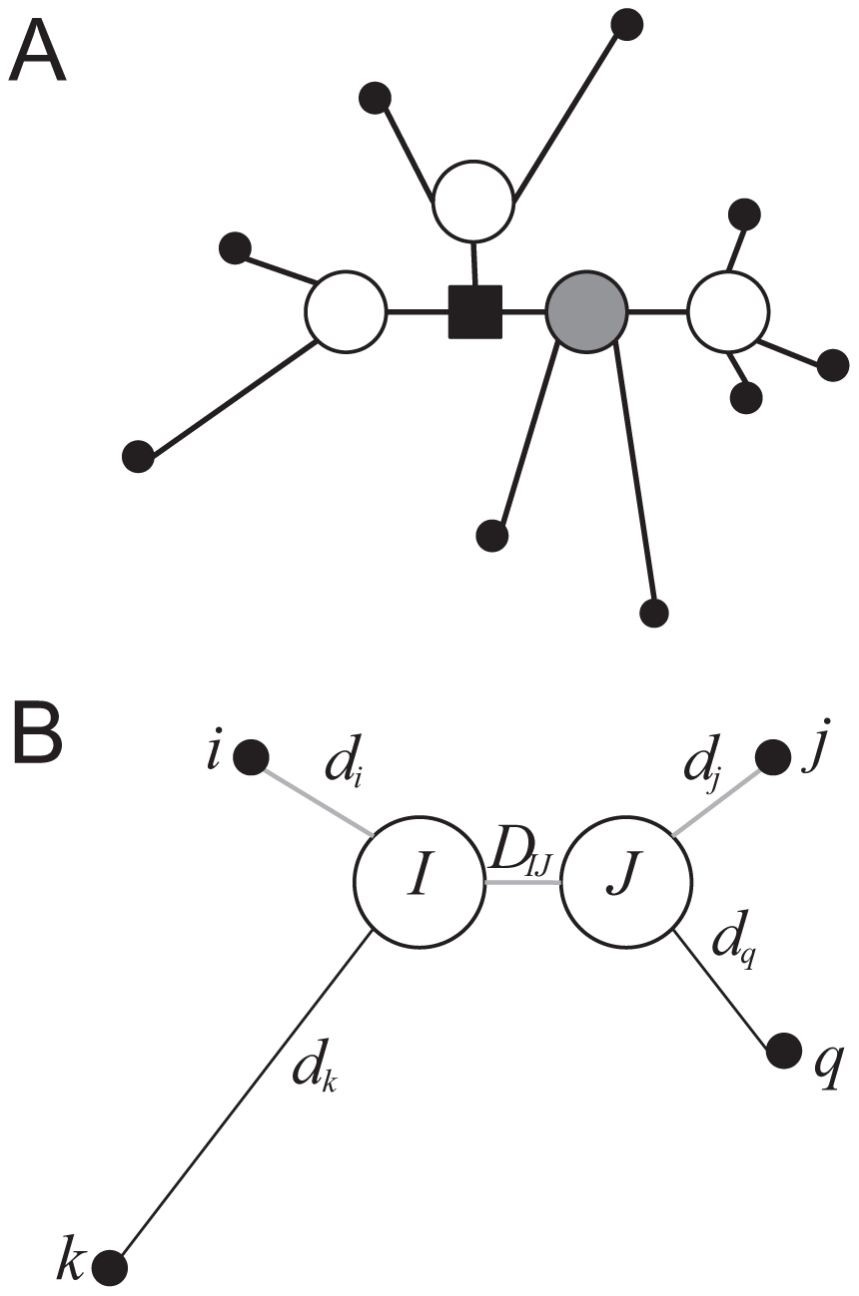
A) Graph illustrating the AMOVA modelling of the genetic relationships of individuals. Each individual, coloured as black vertex, connects to a vertex of type population, which can be either a non-admixed population if it is connected to a single group vertex (black square) or an admixed population if the population vertex is connected to more than one population/group. B) A simple two-population model. The vertex group between the two populations has been removed for clarity. The distance of each specimen to its own population (d.) can be larger than the distance between populations (D_IJ_) to the extent, that based on distances between individuals (D_ij_), individual k would be a single node and individual i would be clustered with individuals j and q.

Here we attempt to reduce the within-population variation and maximize the between-population variation without *a priori* knowledge of the clusters (that is, only using individual pairwise distances) and without any distance restriction. We do this by transforming the genetic distance D matrix into a new dissimilarity one V, where V_ij_ = V_ji_ = var[d_i._–d_j._] taking into account that d_ii_ – d_ij_ and d_jj_ – d_ji_ are excluded during the variance computation. The rationale for proposing the V matrix transformation is as follows:

Following the AMOVA framework, individual relationships are modelled using a list colouring of graph [38], so each vertex can be either assigned to an individual, a non-admixed population, an admixed population, or a group of populations (see Figure 1 A); therefore, for a pair of individuals i, j, the distance d_i,j_ can be decomposed in within- and between-population distances (see Figure 1B):
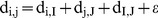
(5)


where d_i,I_ is the distance of individual i to his group I (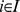), d_j,J_ is the distance of individual j to his group J (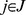), d_I,J_ is the distance between group I and J and ε is a random error in the estimation of any d_.,._ which we assume follows a normal distribution and is identical for all the individual pairwise distances (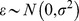). If i and j share the same adjacency vertex (i.e. 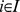 and 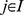), then:
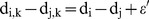
(6)

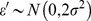
(7)


for any individual k (k≠i; k≠j). The mean of the difference between distances is then:
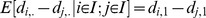
(8)


with expected variance:
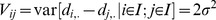
(9)


Therefore, the variance of the difference of distances for a given pair of individuals from the same group to all the other individuals becomes independent of the distance of each individual to his group, and it is the same for all the elements of the group. In contrast, it can be expected that the distances between individuals from different populations will depend on the topology of the graph and the number of individuals that belong to the same population. For example, consider the simplest case of a graph of two populations (Figure 1B); if i and j do not share the same adjacency vertex (i.e. they are from a different populations), V_ij_ becomes:
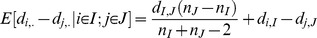
(10)


And
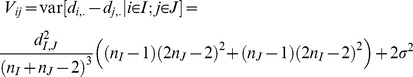
(11)


Where 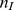 is the number of individuals that belong to population I and 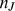 is the number of individuals that belong to population J. In this case, the variance of the difference of distances includes an additional term to the error in the estimation proportional to the distance between the two groups and their respective sample sizes. As previously, the within-population variance (i.e. the distance of the individual to his population) is cancelled, which can therefore improve the detection of population differentiation. If 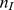 = 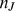, it can be seen that V_ij_ = d^2^_I,J_ + 2σ^2^. Also, notice that if 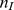 = 1 or 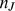 = 1, then V_ij_ = 2σ^2^. Therefore, in this example population differentiation could only be detected by this statistic when there are at least two individuals in each population so that the distance of each individual to his population can be estimated.

The pseudocode for computing V is provided in Text S1.

### Genetic algorithm for exploring the solution space

The AMOVA framework has been previously applied to identifying the best genetically homogeneous sets of geographically related populations [39] by trying to maximize the amount of genetic differentiation among groups of populations (conversely minimizing the variance within groups of populations). Since exploring the entire solution space is unfeasible even for a reduced number of populations, Dupanloup et al. [39] applied a simulated annealing algorithm. The method was devised to detect spatial barriers between already defined populations. However, a similar heuristic approach can also be applied for clustering individuals into populations, rather than populations into groups of populations. In particular, we propose to use a continuous genetic algorithm [40] with Crossover Pair SubClusterSwap_TWO_NEW [41] movement in order to explore the space of possible combinations and recover the optimal (or suboptimal) combination that maximizes the SS(AP) statistic (conversely minimizes SS(WP); see Text S1).

### Computer simulations

In order to test the V matrix transformation in a known graph model, we performed simulations on four populations of 10 individuals each, modelling a situation of three parental populations and one admixed population (see Figure S1). In each simulation, we varied at random the distance of each individual to its population, the distances between populations, and whether the distances of the individuals to their populations were larger than the distances between populations. We performed 1000 simulations for each of the 8 possible combinations, and for each simulation computed the distance between each pair of individuals according to formula (5), including an error term following a normal distribution with mean  = 0 and standard deviation  = 0.05; for each simulation the D and V matrix and the percentage of SS(AP) explained was computed.

**Figure 2:**
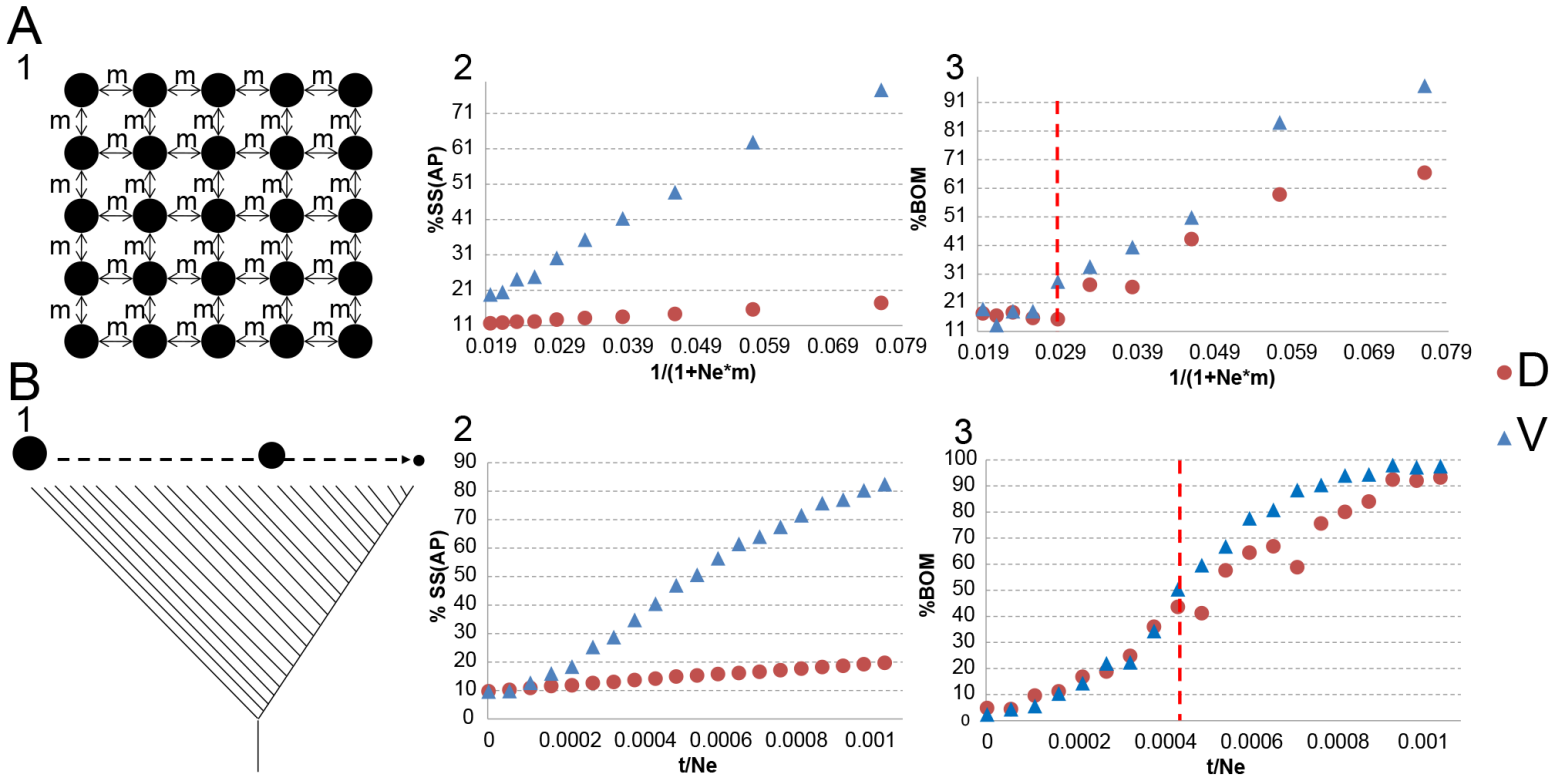
Demographic scenarios used to test the performance of V and D matrices. Each simulation consists of 100,000 randomly ascertained SNPs (see Figure S3 for simulations with 10,000 SNPs) simulated with ms software in 25 populations (10 diploid individuals in each population). A) A.1) 2-D stepping stone demographic model implemented in the simulations. Each population exchanges a fraction of m migrants with the neighbour populations each generation. A.2) Amount of variation explained among populations by using either the D or V matrix in simulated against the inverse of the scaled amount of migrants by generation [57]. A.3) Percentage of best genetic-matching partners (best overall match (BOM)) in the same (sub)population depending on whether the matrix V or D is used. The red dashed line indicates the simulation when the BOM computed from V matrix > BOM computed with D matrix. B) B.1) A sequential split demographic scenario. Each t generations the youngest population (at the right of the plot) splits into two. One remains in the same place and the new one moves to a new position at the right, decreasing its effective population size proportionally to the number of already conducted sequential splits. B.2) Percentage of SS(AP) respect to SS(T) by using either the D or the V matrix against the scaled time of split by Ne. B.3) Percentage of best genetic-matching partners (best overall match (BOM)) in the same (sub)population depending on whether the matrix V or D is used. The red dashed line indicates the simulation when the BOM computed from V matrix > BOM computed with D matrix.

We also conducted two sets of simulations of increasing demographic complexity to check whether V is more sensitive for detecting population substructure than D and to analyse to what extent the use of V improves the geographic sampling location prediction compared to the use of D. Both demographic models were implemented with the ms software [42] and simulated 25 populations, 10 diploid individuals per population (that is, 20 chromosomes per population) and either 10,000 or 100,000 independent SNPs sampled from fragments of 50 kb and assuming a mutation rate of 2.5*10^−8^ per nucleotide and generation [43]. In the first demographic scenario, we model the colonization of a one-dimensional space from a starting founder population by splitting the youngest population in two new ones every t generations [44] (see Figure 2A and Text S1 for details). The second scenario considers spatial structure and migration between neighbour populations following an isolation by distance model [13] (see Figure 2B and Text S1). For each simulated dataset, the sensitivity of D and V towards the real sampling location was quantified by means of SS(AP)/SS(T). We further analysed the performance of V for improving the percentage of best genetic-matching partners in the same population by computing the percentage of BOM.

### European genetic dataset

We used a previously published dataset comprising 309,790 SNPs and 2,457 individuals from 23 European subpopulations genotyped with the Affymetrix 250K Xba and 250K Sty SNP microarrays, [21] (see Table S1). Previous data cleaning of that dataset included removing individuals showing a higher or smaller genetic differentiation compared to the rest of the individuals from the same subpopulation, and excluding SNPs showing a statistically significant HWE deviation in at least one subpopulation (see [21] for a complete description of the data cleaning procedure). Since most of the applied methods assume linkage equilibrium among SNPs, a Linkage Disequilibrium (LD) pruned SNP subset of 133,363 was computed with plink software [45] with the default *plink --indep 50 5 2* command, and was used for method-comparison analyses. Also, since multivariate techniques such as PCA have shown that unequal sample size can affect the outcome [46], all analyses were performed twice, once considering the original sample size and once considering 19 sample sites or subpopulations with a sample size of 40 individuals (after excluding Lisbon-Portugal, Dublin-Ireland, Budapest-Hungary and Bucharest-Romania) polymorphic at 124,134 SNPs. We attempted to apply five of the previously proposed methods for inferring groups of genetically homogeneous individuals (for example, see [47]) to this dataset. When not included in the original algorithm, we applied the algorithm Mclust [48] to obtain the clusters. This algorithm assigns individuals to clusters by fitting multivariate normal distributions using the coordinates of the proposed dimensions and proposes the best clustering based on the Bayesian Information Criterion (BIC). Mclust has been put forward as a clustering algorithm for the output of Principal Component Analysis using genetic data [49].

The first analysis consisted of a Classical Multidimensional Scaling (MDS;[9]) performed using either the D or the V distance matrix between pairs of individuals using the cmdscale function of R statistical package [50], and adding a constant to avoid negative eigenvalues [51]; Mclust clustering was performed using the first 10 dimensions, and setting the number of clusters from 1 to 60. The second analysis consisted of a spatial ancestry analysis (SPA)[12] conducted to infer the geographic ancestry of each individual in two spatial dimensions. Clusters of individuals were then inferred by means of Mclust using the proposed SPA coordinates, also ranging from 1 to 60. The third analysis was performed with the *clusterGem* algorithm implemented in the GemTools package which uses spectral graph theory to propose clusters of individuals [52]. Recently, a new software called LOCO-LD [53] has been proposed for estimating the geographic locations of a set of individuals. Similar in essence to SPA (i.e. for each SNP it is assumed that there is an allelic gradient), LOCO-LD additionally incorporates LD patterns into the model, which has been suggested to improve ancestry detection. However, the fact that it necessarily requires a training dataset where the localization of some individuals is known *a priori* (personal communication with the authors) has precluded its use for comparative purposes, as all the other used algorithms are unsupervised. We also aimed to run fineSTRUCTURE, another software that uses LD patterns [26], on the same dataset, after phasing it with the Beagle software [54]. However, computing the shared chunk matrix of all individuals with the default parameters of ChromoPainting [26] turned out to be extremely computationally intensive, even after splitting the genome into chromosomes for parallel computing. As an example, chromosome 22, the smallest human chromosome comprising only 3,698 SNPs in this dataset, has a computational complexity according to the ChromoPainter manual of 96,589,584,000 steps for only one E-M iteration. The authors of this software reported in the ChromoPainter manual computation times of 2-3 hours for a computational complexity of 115,543,296 steps using a computer of similar characteristics as the one we used here (8 cpus, 24 GB of RAM). Therefore, it can be expected that the computational time for this chromosome is going to be ∼83*(2 to 3) hours. Given that the authors suggest to run ChromoPainter considering different numbers of E-M iterations and parameters, running all 22 chromosomes of this dataset appears beyond reasonable practicability with the computer resources available. Because of this, we decided to exclude this software from comparison.

Pie map plots were constructed for each method and each proposed clustering using the R packages *map* and *mapplots*.

### Estimation of the sampling site differentiation based on proposed genetic clusters

The Cramer's V value [55] was used for summarizing the goodness of fitness between the proposed clusters and the labelled population origin of the individuals. Cramer's V is a classical measure of association of two variables in a contingency table and is defined as:
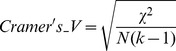
(12)


where χ^2^ is the chi-squared value from Pearson's chi-squared test, N is the total of observations, k is the number of rows or the number of columns if less than the number of rows. Cramer's V ranges from 0, which corresponds to random assignment of the individuals of each population to the different clusters, to 1, meaning that each proposed individual cluster perfectly matches one population.

Also, in order to quantify how well the genetic clusters proposed by each method differentiate each sampling location or subpopulation from all the others, we computed the Informativeness of Ancestry (I_n_) statistic [37] between each pair of sampling locations using the obtained frequency of the proposed clusters by each method:
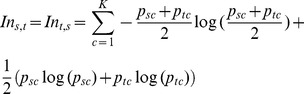
(13)


Where K is the number of proposed clusters, p_sc_ is the frequency of the cluster c in sampling location s and p_tc_ is the frequency of the cluster c in sampling location t.

I_n_ ranges between 0 (i.e. the proposed clusters cannot distinguish individuals from the two populations) to log(2), indicating that the two sampling locations are perfectly differentiable based on the proposed clusters. Therefore, if a sampling location is perfectly differentiated from any other sampling location based on the proposed clusters, the minimum value that is going to be obtained for all the possible (sub)population comparisons of that particular sampling site is log(2). In contrast, if the sampling location is identical based on the proposed clusters to at least one of the other sampling locations, the minimum I_n_ value is going to be 0.

The complete methodological pipeline is depicted in Figure S2

## Results/Discussion

In the present study, we propose the use of V, the variance in the difference of distances between two individuals to all the other tested individuals, in order to homogenize and minimize the genetic distance within each (sub)population, thus enhancing the between-(sub)population genetic differentiation. This matrix conversion, coupled to a genetic algorithm that uses the AMOVA framework, is then employed to highlight the presence of hidden genetic relationships between individuals and provide clusters of genetically related individuals. We have named this newly developed approach Genetic Algorithm for Genetic Ancestry (GAGA).

### Testing the new approach by means of computer simulations

We started comparing V and D matrices in explaining the between-population variation in a simple case modelling four populations under different scenarios of distances between individuals and populations. As can be seen in Figure S1, SS(AP) computed with the D matrix strongly varies depending on which distance model assumptions are applied. In contrast, the SS(AP) values obtained with the V matrix are close to 1 in all cases, and are in all the cases larger than those obtained for the same simulation with the D matrix.

Next, we analysed the behaviour of the V and D matrix in genetic data by means of extensive simulations using two of the most commonly applied models in human populations, considering either 10,000 SNPs ( see Figure S3) or 100,000 SNPs (see Figure 2). In the two-dimensional stepping-stone grid model the amount of genetic differentiation between populations increased proportionally to the decrease in the number of migrants among neighbour populations when using either D or V matrix, regardless of the number of considered SNPs (see Figure 2A.2). Nevertheless, the between-population differentiation increased much faster in the case of V than in the case of D and even faster when simulating 100,000 SNPs (see Figure 2 and Figure S3; Wilcoxon signed paired rank test p-value between SS(AP) estimated from V matrix with either 10,000 SNPs or 100,000 SNPs  =  0.001953). In contrast, SS(AP) values estimated with the D matrix were similar, independently of the number of considered SNPs (Wilcoxon signed paired rank test p-value between SS(AP) estimated from D matrix with either 10,000 SNPs or 100,000 SNPs  =  0.4316). A similar trend of results, both for the V matrix and the D matrix, was observed when simulating the data under the sequential split model and increasing the time of separation between populations (see Figure 2B.2). Furthermore, the percentage of BOM from the same sampling population increases when the migration rate decreases (in the case of the stepping-stone model) and the time of split increases (in the case of the sequential split model), independently of the number of SNPs or type of considered distance matrix (see Figure 2A.3 and 2B.3). However, the percentage of BOM from the same sampled population increases faster when using V than when using D after a certain parameter threshold in both models (see Figure 2.A.3 and Figure 2.B.3). Furthermore, this threshold depends on the number of considered SNPs (see Figure S3): a smaller migration rate for the sequential split model and a larger time of population split for the stepping stone model is required in order to detect differences in the percentage of BOM from V or D matrix using 10,000 SNPs compared to when using 100,000 SNPs.

Overall, our simulation experiments demonstrate that V can be used to detect further genetic-geographic population substructure in the cases where the amount of genetic differentiation is particularly small compared to within each population, such as is expected and partly known already in human populations from the European continent.

### Application of the V matrix on human genome-wide data from Europe

Given these promising results obtained in the computer simulations, we applied our newly developed approach to a previously collected dataset comprising 2,457 individuals from 23 European subpopulations using 133,363 LD pruned genome-wide SNPs [21]. We first observed that the mean distance T1 of each individual to all the other individuals collected at the same geographic site (i.e. belonging to the same subpopulation) was 0.331 (95% CI from 0.322 to 0.342). Thirty-three percent of the individuals showed a mean T1 distance to their sampling population >1/3, suggesting that they belonged to a different random mating population [31]. Moreover, this proportion was not constant among European subpopulations (ranging from 0% in Budapest-Hungary to 63% in Madrid-Spain; see Table S2, two sided Fisher exact test p value < 0.0005 after 2000 replicates), indicating that some European subpopulations are more genetically heterogeneous than others. The percentage of individuals with BOM in the same subpopulation using the T1 matrix was 25.93%, a value similar to the one obtained previously when using Identical By State distance between pairs of individuals [24]. This value ranged from 0% in Bucharest-Romania, Copenhagen-Denmark, Lyon-France, Prague-Czech Republic and Warsaw-Poland to 78.7% in Helsinki-Finland (Table S3). In contrast, the BOM computed from the V distance matrix increased to 52.83%, ranging from 6% in Lyon to 97.87% in Helsinki (see Table S4). This improvement is much higher than the one observed in the simulated datasets for BOM of 20% computed with the D matrix. Furthermore, the SS(AP) was estimated to be 1.62% when using the D matrix, while it increased to 7.98% when using the V matrix. Hence, also when applied to real genomic data our newly developed approach revealed increased genetic population differentiation.

### Classification improvement when applying the V matrix compared to the D matrix

**Figure 3:**
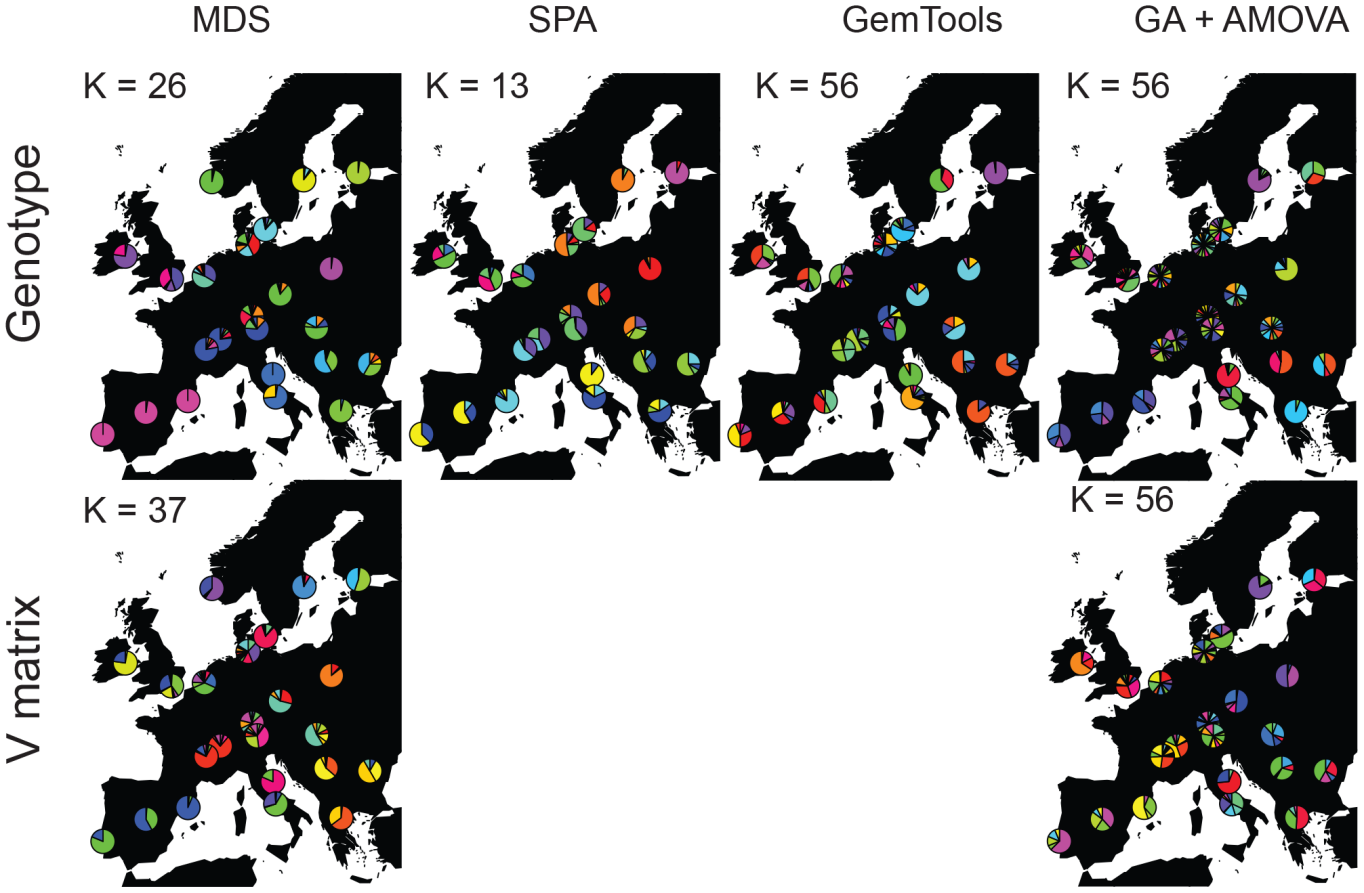
Pie maps of 2,457 European individuals from 23 sampling subpopulations from across Europe analysed at 133,363 Linkage Disequilibrium (LD) pruned SNPs according to their genetic relationships using: Classical Multidimensional Scaling (MDS) + Mclust analysis using the D distance matrix based on the T1 (Genotype) statistic; MDS + Mclust analysis using the distance matrix with the transformed V matrix; SPA + Mclust analysis using the original genotype data; GemTools analysis using the original genotype data; genetic algorithm + AMOVA using the original D matrix; genetic algorithm + AMOVA using the V matrix.

**Figure 4:**
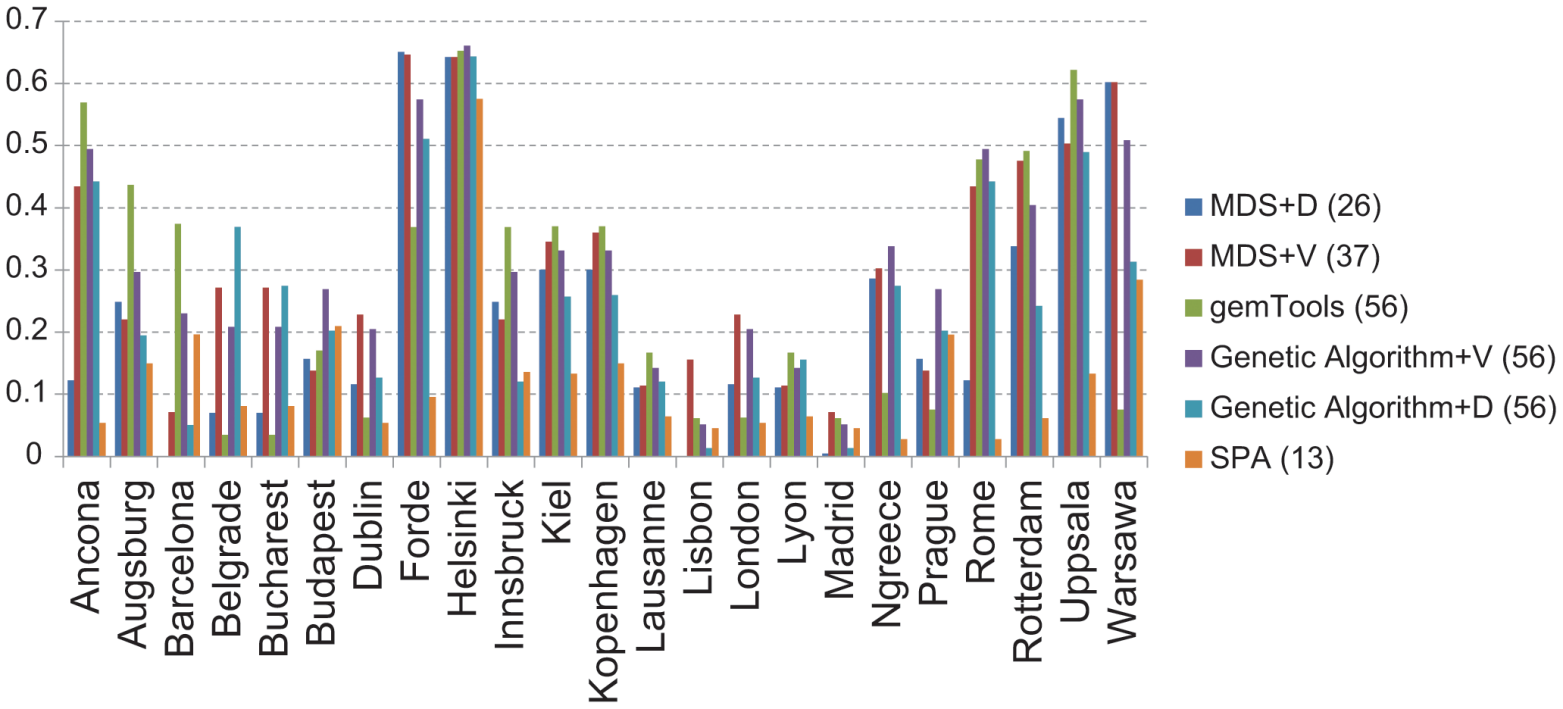
Minimum amount of genetic differentiation of each of the 23 European subpopulation against the others estimated from the proposed clusters of each method using a genome-wide dataset of 133,363 LD pruned genome-wide autosomal SNPs from 2,457 individuals [21].

We further focused on studying to which extent unsupervised clusters of individuals inferred from the genetic data would match the geographic site of their sampling origin or subpopulation (see Table 1). In the case of MDS, the first two dimensions using the D matrix and considering all the individuals explained 0.733% of the total variance (see Figure S4), 2% when considering an equal sample size of 40 individuals per subpopulation. In contrast, the first two dimensions of the MDS using V and all the individuals explained 15.133% of the total variance, 20.64 times more, and increased to 30.45% when using unbiased sample size among populations. These results supports that the V transformation reduces the amount of non-shared (i.e. particular of each individual) variation, and highlights the differences among groups of individuals. The best supported clustering by Mclust using the first 10 MDS dimensions using the D matrix was 26 genetic clusters (Figure 3A). Cramer’s V statistic between the proposed clusters and the sampling sites or subpopulations was 0.655. The average amount of minimum sampling site differentiation based on these 26 clusters was 0.231, with Helsinki-Finland being the mostly differentiated of all European subpopulations considered (I_n_ = 0.642, see Figure 4) and Lisbon-Portugal, Madrid-Spain and Barcelona-Spain appearing as non-distinguishable from each other (I_n_ = 0). In contrast, Mclust using the first 10 MDS dimensions from the V matrix proposed 37 different genetic clusters, all the populations sharing at least one of the proposed clusters (see Figure 3B). Cramer’s V increased to 0.71, and the average I_n_ increased to 0.304, again suggesting that V provides a better population sampling resolution than the original D matrix. The strongest improvement in European subpopulation differentiation was observed in Ancona-Italy (I_n_ using the D matrix (I_n_–D)  = 0.122 compared to In using the V matrix (I_n_–V)  = 0.434) and Rome-Italy (I_n_–D = 0.122 to I_n_–V = 434). Running Mclust on SPA based on the original genotype matrix suggested 13 clusters (Figure 3C); the average amount of subpopulation differentiation provided by these genetic clusters was quite poor (average I_n_ = 0.127; see Figure 4), and none of the sampling subpopulations improved their differentiation compared to all the other methods. Nevertheless, it must be taken into account that these results are not directly comparable, since SPA models the observed data in a very limited number of dimensions (two in our case), whereas MDS+Mclust analyses were based on 10 dimensions. Indeed, the MDS+Mclust analysis using the first two dimensions provided similar results to the ones observed with SPA (results not shown). GemTools analysis proposed 56 different genetic clusters (see Figure 3D). However, despite this increase in the number of proposed clusters, the average minimum differentiation among subpopulations (average I_n_ = 0.268) was smaller than the one obtained when running MDS-V+Mclust. Compared to MDS-V+Mclust, the proposed clusters by GemTools increased the differentiation of Barcelona-Spain, Ancona-Italy, Augsburg-Germany, and Innsbruck-Austria but reduced it in Belgrade-Serbia, Bucharest-Romania, North Greece, Forde-Norway, and particularly in Warsaw-Poland (see Figure 4). We used the genetic algorithm maximizing AMOVA's SS(AP) statistic either with the D or the V matrix (the latest comprising the GAGA approach) to the same genetic dataset, setting K = 56 allowed clusters (see Figure S5 for results using K = 2, 5, 10, 15 and 23 with V matrix), the same number of clusters as identified by GemTools (see Figure 3E). The average amount of minimum genetic differentiation of the proposed clusters by the genetic algorithm + D matrix was I_n_ = 0.254, the second worse value after MDS-D+Mclust. Only in the case of Belgrade there was an improvement compared to all the other methods (see Figure 4). In contrast to these results, when the genetic algorithm uses the V matrix, the average differentiation among European subpopulations increased to I_n_ = 0.316, the largest value of all the applied methods. Hence, GAGA was able to increase the geographic resolution compared to other methods. Furthermore, in the case of Budapest-Hungary, North Greece, Helsinki-Finland, Prague-Czech Republic and Rome-Italy (Figure 4), GAGA provides the best values of subpopulation differentiation in this European genomic dataset.

**Table 1:** Estimated association by means of Cramer’s V and mean minimum population informativeness differentiation between proposed clusters by different clustering methods and (sub)population sampling origin using: all the samples (2457 individuals) and populations (23), 40 samples per population in 19 populations and all the samples from 19 populations (see Materials and Methods).

Method	All samples and populations			40 samples per population, 19 populations			All samples, 19 populations		
	Clusters	Cramer’s V	Mean min I_n_	Clusters	Cramer’s V	Mean min I_n_	Clusters	Cramer’s V	Mean min I_n_
MDS+D + Mclust	26	0.655	0.231	19	0.753	0.291	33	0.752	0.337
MDS+V+ Mclust	37	0.71	0.304	17	0.793	0.273	28	0.767	0.343
SPA+Mclust	13	0.621	0.127	8	0.8	0.120	11	0.604	0.107
GemTools	56	0.685	0.268	25	0.768	0.375	56	0.781	0.385
Genetic algorithm+D	56	0.618	0.254	56	0.66	0.300	56	0.663	0.294
GAGA	56	0.701	0.316	56	0.745	0.315	56	0.763	0.382

We further analysed the effect of different sample size in the outcome of the different methods. We repeated all the analyses with a subset of 19 populations (after excluding Lisbon-Portugal, Dublin-Ireland, Budapest-Hungary and Bucharest- Romania) with equal sample size of 40 individuals. The percentage of closest genetic neighbours in the same (sub)population is similar to the ones when considering all the individuals (BOM = 58.03% for the V matrix, 22.37% for the D matrix). Nevertheless, the association between the proposed clusters and the (sub)population samples increases in all the methods. This is particularly pronounced in the case of GemTools (see Table 1). We wondered whether this difference in performance of GemTools is due to the excluded four populations and/or to the use of equal sample sizes, so we performed all the analyses considering the same subset of 19 subpopulations but with their original sample size. The values of minimum informativeness differentiation of GemTools increased to 0.385, thus suggesting the influence of the four excluded populations in the final results.

### Conclusions

We have described a new matrix distance transformation that tends to minimize the within-population variance without knowing *a priori* the (sub)populations, and have shown, by means of computer simulations and application to real European genetic data, that this new approach improves the differentiation among (sub)populations compared to the original distance matrix. A practical result of our analyses is that this matrix transformation improves the output of MDS, both at the level of explained variance and resolution, as well as from the AMOVA estimations. In the present paper we show that GAGA performs reasonably well when using the K proposed by GemTools. One could also consider estimating the K based on parameterized Gaussian mixture models [56] such as implemented in Mclust. Nevertheless, the choice of K is rather arbitrary depending on the required resolution and subject of further study. Most importantly, our findings of previously undetected fine-scale human population substructure down to the level of sampling sites or subpopulations within Europe, has important implications for various basic and applied fields of life science. With relevance for genetic epidemiology, our results suggest that the genetic homogeneity detection desired in case-control studies should be preferably established by analyzing the relationships of pairs of individuals in the context of all other individuals tested, rather than by analyzing how genetically similar individuals are, as usually done. The GAGA approach we introduce here is now available for application to all types of genetic data. The GAGA algorithm was implemented in JAVA (Sun Microsystems) and is publically available for widespread use at http://www.erasmusmc.nl/fmb/resources/GAGA.

## Supporting Information

Figure S1 A) Model of three parental populations and one admixed population, each one with 10 individuals (black dots, only two individuals per population are shown in the graph). Eight different possible situations where considered. The edge of each individual to his adjacency population vertex were either all of the same length (Individual Distance Constant, IDC) or of variable length (Individual Distance Not Constant, IDNC). The edges connecting two populations were either all of the same length (Group Distance Constant, GDC) or of variable length (Group Distance Not Constant, GDNC) and also larger than the minimum distance of any individual to his population (GDLI) or smaller (GDSI). For each possible combination, 1000 simulations were conducted. The edge distance of an individual to the adjacency vertex population was randomly modelled using a uniform distribution U(0.5, 1). The assumed error in the estimation was computed following a Normal distribution N(0, 0.05). The distance between adjacent populations was simulated from a uniform distribution with parameters U(m, 1) if the distance was larger than the minimum individual distance to his population (m) or U(0,m) if the distance between two adjacent populations was smaller. B) Boxplot of the SS(AP)/SS(T) computed for each of the 1000 simulations conducted for each of the eight possible combinations. In grey, SS(AP)/SS(T) estimations considering the original Distance matrix, computed as the path between two points given their simulated distances to their population of origin and the distances between populations. In black, SS(AP)/SS(T) estimations considering the transformed V matrix, computed out of the original Distance matrix. As can be seen, in all the simulated cases SS(AP)/SS(T) of the V matrix is > SS(AP)/SS(T) of the D matrix.
(EPS)

Figure S2 Analysis pipeline applied to the genome-wide data from 2,457 individuals from 23 European subpopulations [21]. Starting from the genotype matrix of n = 2,457 individuals by m = 133,363 LD pruned loci, a distance matrix D is computed using T1 statistic or similar ones (procedure 1). The distance matrix is then used to perform a MDS analysis (procedure 2), resulting in a set of MDS coordinates in a reduced Euclidean space. Applying clustering algorithms, such as Mclust, on the MDS coordinates by supplying an arbitrary number of clusters, k, will assign all individuals to k clusters (procedure 3). This clustering configuration can be evaluated for concordance with their true population sampling origin labels using cross-tabulations (procedure 4) by means of, for example, minimum Informativeness of ancestry, which gives a single numeric value for each population between 0 and log(2) with a larger value for a higher population differentiation (procedure 5). In parallel, either GemTools or SPA is applied to the original genotype matrix. In the case of SPA, Mclust is applied to identify clusters of individuals (procedure 4). Our new algorithm, GAGA, starts by transforming the D matrix into the V matrix (procedure 7). This step highlights the genetic differentiation among (the *a priori* unknown) (sub)populations. A genetic algorithm is then applied to search for the optimal clustering configuration (procedure 8). The clustering results from GAGA can also be compared with those from other algorithms such as MDS and SPA through procedures 4 and 5.
(EPS)

Figure S3 Demographic scenarios used to test the performance of V and D matrices. Each simulation consists of 10,000 randomly ascertained SNPs (see Figure 2 for simulations with 100,000 SNPs) simulated with ms software in 25 populations (10 diploid individuals in each population). A) A.1) 2-D stepping stone demographic model implemented in the simulations. Each population exchanges a fraction of m migrants with the neighbor populations each generation. A.2) Amount of variation explained among populations by either the D or V matrix in simulated against the inverse of the scaled amount of migrants by generation [57]. A.3) Percentage of the best genetic-matching partner (best overall match (BOM)) in the same (sub)population depending on whether the matrix V or D is used. B) B.1) A sequential split demographic scenario. Each t generations the youngest population (at the right of the plot) splits into two. One remains in the same place and the new one moves to a new position at the right, decreasing its effective population size proportionally to the number of already conducted sequential splits. B.2) Percentage of SS(AP) respect to SS(T) when using either the D or the V matrix against the scaled time of split by Ne. B.3) Percentage of closest genetic neighbor (best overall match (BOM)) in the same (sub)population depending on whether the matrix V or D is used.
(EPS)

Figure S4 Percentage of variation explained by each eigenvalue from a classical Multidimensional Scaling analysis when using the D (based on the T1 statistic) or the transformed V distance matrix on 2,457 European individuals sampled at 133,363 Linkage Disequilibrium (LD) pruned SNPs.
(EPS)

Figure S5 Best proposed clusters using GAGA setting K = 2, 5, 10, 15 and 23 on 2,457 European individuals sampled at 133,363 Linkage Disequilibrium (LD) pruned SNPs.
(EPS)

Table S1 2457 European samples from 23 sampling locations/subpopulations used in the study after the data cleaning performed in [21]. Underlined populations were excluded from the analyses considering equal sample size.
(DOCX)

Table S2 Counts of European individuals showing a mean D < 1/3 (indicating more relatedness to the population than the expected by random mating) and mean D > 1/3 (indicating that the individual is on average from a different random mating population than the one where he was sampled).
(DOCX)

Table S3 Table showing the individuals with the best overall genetic match (BOM) in the same population of sampling or in a different population when using the D statistic based on T1 similarity as measure of genetic dissimilarity.
(DOCX)

Table S4 Table showing the individuals with the BOM in the same population of sampling or in a different population when using the V statistic as measure of genetic dissimilarity.
(DOCX)

Text S1 Supplementary information describing the Pseudocode for the Computation of the V matrix, implementation of the Genetic algorithm for exploring the space of solutions and demographic simulations.
(DOC)
