# Supplementary material for: GAGA: A New Algorithm for Genomic Inference of Geographic Ancestry Reveals Fine Level Population Substructure in Europeans
Source: PLoS Comput Biol. 2014 Feb 20;10(2):e1003480. doi: 10.1371/journal.pcbi.1003480 (PMC3930519; doi:10.1371/journal.pcbi.1003480)
Supplement: Table S1 — 2457 European samples from 23 sampling locations/subpopulations used in the study after the data cleaning performed in [21]. Underlined populations were excluded from the analyses considering equal sample size. (DOCX) [file pcbi.1003480.s006.docx]

**Table S1.** 2457 European samples from 23 sampling locations/subpopulations used in the study after the data cleaning performed in [[7](#_ENREF_7)].

| **Population** | **Latitude** | **Longitude** | **Total sample size** |
| --- | --- | --- | --- |
| Ancona | 43.37 | 13.3 | 49 |
| Augsburg | 48.21 | 10.54 | 489 |
| Barcelona | 41.23 | 2.1 | 47 |
| Belgrade | 44.49 | 20.3 | 55 |
| Bucharest | 44.25 | 26.07 | 12 |
| Budapest | 47.27 | 19.06 | 17 |
| Dublin | 53.19 | -6.15 | 35 |
| Forde | 59.36 | 5.28 | 52 |
| Helsinki | 60.1 | 24.56 | 47 |
| Innsbruck | 47.16 | 11.23 | 50 |
| Kiel | 54.14 | 10.04 | 494 |
| Kopenhagen | 55.4 | 12.34 | 59 |
| Lausanne | 46.31 | 6.37 | 133 |
| Lisboa | 38.43 | -9.08 | 16 |
| London | 51.3 | -0.07 | 194 |
| Lyon | 45.46 | 4.5 | 50 |
| Madrid | 40.25 | -3.42 | 81 |
| Ngreece | 40.38 | 22.27 | 51 |
| Prague | 50.04 | 14.28 | 45 |
| Rome | 41.53 | 13.68 | 106 |
| Rotterdam | 51.55 | 4.28 | 280 |
| Uppsala | 59.51 | 17.38 | 46 |
| Warsaw | 52.15 | 21.01 | 49 |
