# Supplementary material for: GAGA: A New Algorithm for Genomic Inference of Geographic Ancestry Reveals Fine Level Population Substructure in Europeans
Source: PLoS Comput Biol. 2014 Feb 20;10(2):e1003480. doi: 10.1371/journal.pcbi.1003480 (PMC3930519; doi:10.1371/journal.pcbi.1003480)
Supplement: Table S2 — Counts of European individuals showing a mean D < 1/3 (indicating more relatedness to the population than the expected by random mating) and mean D > 1/3 (indicating that the individual is on average from a different random mating population than the one where he was sampled). (DOCX) [file pcbi.1003480.s007.docx]

**Table S2**. Counts of European individuals showing a mean D < 1/3 (indicating more relatedness to the population than the expected by random mating) and mean D > 1/3 (indicating that the individual is on average from a different random mating population than the one where he was sampled)

| **Population** | **D<1/3** | **D>1/3** |
| --- | --- | --- |
| Ancona | 40 | 9 |
| Augsburg | 247 | 242 |
| Barcelona | 42 | 5 |
| Belgrade | 33 | 22 |
| Bucharest | 9 | 3 |
| Budapest | 17 | 0 |
| Dublin | 25 | 10 |
| Fnorde | 23 | 29 |
| Helsinki | 26 | 21 |
| Innsbruck | 49 | 1 |
| Kiel | 354 | 140 |
| Kopenhagen | 44 | 15 |
| Lausanne | 106 | 27 |
| Lisboa | 3 | 13 |
| London | 172 | 22 |
| Lyon | 39 | 11 |
| Madrid | 30 | 51 |
| Ngreece | 45 | 6 |
| Prague | 23 | 22 |
| Rome | 45 | 61 |
| Rotterdam | 201 | 79 |
| Uppsala | 40 | 6 |
| Warsaw | 32 | 17 |
