# Supplementary material for: GAGA: A New Algorithm for Genomic Inference of Geographic Ancestry Reveals Fine Level Population Substructure in Europeans
Source: PLoS Comput Biol. 2014 Feb 20;10(2):e1003480. doi: 10.1371/journal.pcbi.1003480 (PMC3930519; doi:10.1371/journal.pcbi.1003480)
Supplement: Table S3 — Table showing the individuals with the best overall genetic match (BOM) in the same population of sampling or in a different population when using the D statistic based on T1 similarity as measure of genetic dissimilarity. (DOCX) [file pcbi.1003480.s008.docx]

**Table S3**. Table showing the individuals with the best overall genetic match (BOM) in the same population of sampling or in a different population when using the D statistic based on T1 similarity as measure of genetic dissimilarity

| Population | BOM same population | BOM in different population |
| --- | --- | --- |
| Ancona | 10 | 39 |
| Augsburg | 115 | 374 |
| Barcelona | 34 | 13 |
| Belgrade | 2 | 53 |
| Bucharest | 0 | 12 |
| Budapest | 2 | 15 |
| Dublin | 1 | 34 |
| Forde | 18 | 34 |
| Helsinki | 37 | 10 |
| Innsbruck | 11 | 39 |
| Kiel | 252 | 242 |
| Kopenhagen | 0 | 59 |
| Lausanne | 4 | 129 |
| Lisboa | 1 | 15 |
| London | 27 | 167 |
| Lyon | 0 | 50 |
| Madrid | 6 | 75 |
| Ngreece | 11 | 40 |
| Prague | 0 | 45 |
| Rome | 5 | 101 |
| Rotterdam | 93 | 187 |
| Uppsala | 8 | 38 |
| Warsaw | 0 | 49 |
