# Supplementary material for: GAGA: A New Algorithm for Genomic Inference of Geographic Ancestry Reveals Fine Level Population Substructure in Europeans
Source: PLoS Comput Biol. 2014 Feb 20;10(2):e1003480. doi: 10.1371/journal.pcbi.1003480 (PMC3930519; doi:10.1371/journal.pcbi.1003480)
Supplement: Table S4 — Table showing the individuals with the BOM in the same population of sampling or in a different population when using the V statistic as measure of genetic dissimilarity. (DOCX) [file pcbi.1003480.s009.docx]

**Table S4**. Table showing the individuals with the BOM in the same population of sampling or in a different population when using the V statistic as measure of genetic dissimilarity

| Population | BOM same population | BOM in different population |
| --- | --- | --- |
| Ancona | 24 | 25 |
| Augsburg | 253 | 236 |
| Barcelona | 23 | 24 |
| Belgrade | 34 | 21 |
| Bucharest | 2 | 10 |
| Budapest | 3 | 14 |
| Dublin | 6 | 29 |
| Forde | 33 | 19 |
| Helsinki | 46 | 1 |
| Innsbruck | 8 | 42 |
| Kiel | 271 | 223 |
| Kopenhagen | 8 | 51 |
| Lausanne | 87 | 46 |
| Lisboa | 4 | 12 |
| London | 85 | 109 |
| Lyon | 3 | 47 |
| Madrid | 45 | 36 |
| Ngreece | 39 | 12 |
| Prague | 5 | 40 |
| Rome | 83 | 23 |
| Rotterdam | 186 | 94 |
| Uppsala | 25 | 21 |
| Warsaw | 25 | 24 |
