# Supplementary material for: GAGA: A New Algorithm for Genomic Inference of Geographic Ancestry Reveals Fine Level Population Substructure in Europeans
Source: PLoS Comput Biol. 2014 Feb 20;10(2):e1003480. doi: 10.1371/journal.pcbi.1003480 (PMC3930519; doi:10.1371/journal.pcbi.1003480)
Supplement: Text S1 — Supplementary information describing the Pseudocode for the Computation of the V matrix, implementation of the Genetic algorithm for exploring the space of solutions and demographic simulations. (DOC) [file pcbi.1003480.s010.doc]

# Text S1

**Pseudocode for the Computation of the V matrix**

**getV(D)**

**begin** "*get a V matrix from a D matrix*"

V := matrix[nrow(D),ncol(D)]

i := 1

**while** i!= (nrow(D) - 1) **do**

j := i + 1

**while** j!=nrow(D) **do**

V[i,j] := var(D[-c(i,j),i]-D[-c(i,j),j]) *"compute variance of the difference, excluding the value of the cell c(i,j)"*

V[j,i] := V[i,j]

j := j + 1

**end**

i := i+1

**end**

**end**

**Implementation of the Genetic algorithm for exploring the space of solutions**

A genetic algorithm emulates the biological process of natural selection of random solutions in order to improve the population of answers. Each possible solution is called chromosome, the variables of interest are called loci, and the proximity to the optimal solution is defined as the fitness of the chromosome. The algorithm starts with an initial set of random clustering solutions. Each generation is divided in four steps: i) computation of the fitness of each chromosome (SS(AP) value given the proposed clusters), ii) ascertaining a so-called elite chromosomes (selection step) with the largest SS(AP) values and excluding the rest, iii) producing new individual cluster solutions from the combination of the previously ascertained solutions (mating & recombination step), and iv) proposing at random new individual cluster solutions (mutation step). Selection and mating keep the best answers in the current population of answers. The mutation and recombination process allows for a random walking through all the possible set of answers in order to prevent getting staked in a local minimum. The parameters to set in the genetic algorithm are: number of chromosomes, selection coefficient, type of crossover, mutation frequency and number of iterations without updating the solution of the best answer.

We implemented the genetic algorithm with a population of 100 chromosomes per generation. Each chromosome contained an initially proposed cluster solution to each of the n individuals to be classified. The initialization of each chromosome was performed by assigning each individual to one of the K possible groups at random (i.e. no prior knowledge of the clusters was assumed). Selection coefficient varied in time: we used a series of selection-relaxation pulses (100 generations under strong elite selection, 1000 under a relaxed selection) in order to reduce the probability of remaining at local minimum. Elitism was set to 0.9 (equivalent to retaining each generation the 10 best clustering solutions out of the 100) during the strong selection process, and 0.5 (50 best clustering solutions are retained out of the 100), during the relaxation step. The probability of matting of one chromosome was inversely proportional to its position in the array of solutions after sorting by the associated SS(AP) value [40]. The Crossover Pair SubClusterSwap_TWO_NEW [41] movement was used to recombine two chromosomes. This recombination movement has been proposed for the particular case of clustering, where cluster labels are exchangeable. Similarly, we considered two types of mutation movements [41]. The first type consists of taking one individual of the chromosome at random and assigning it to a different cluster from which it belongs to. The second movement consists of merging the individuals of one cluster taken at random with another cluster taken at random. Then, one individual from a cluster that is different from the two implied in the merging is taken at random and assigned to the cluster that was emptied.

Each run of the genetic algorithm finished when there was no improvement of fitness in the best solution (the one with the maximum SS(AP) value out of the 100 chromosomes) after 1000 generations; the best solution maximizing SS(AP) out of ten runs was ascertained as the best one.

**Simulations**

**Sequential population split (Model one)**

In each simulation, the effective population size of the founder population was set to 2,000 chromosomes (that is, 1000 diploid individuals) and $\alpha=-0.036p+1$, *p* being the integer number of current population split ($p\in[1,24]$). With these settings, the founder population had a Ne = 2,000 chromosomes and the youngest population a Ne = 193 chromosomes in each simulation. The time of split (t) differed among simulations from t = 0.005 to t = 2.

One of the descendent populations remains in the place of the parental population with the same effective population size (*Ne_parental_*) whereas the other has a new *Ne* = *Ne_founder_* * α(α < 1) in order to emulate the founder effect observed in human populations [45] and moves to a new position away from the founder and parental populations.

The ms command for t = 0.5 is:

*ms 500 10000 -t 10.0 -I 25 20 20 20 20 20 20 20 20 20 20 20 20 20 20 20 20 20 20 20 20 20 20 20 20 20 -n 1 1.0 -n 2 0.9625 -n 3 0.925 -n 4 0.8875 -n 5 0.85 -n 6 0.8125 -n 7 0.775 -n 8 0.7375 -n 9 0.7 -n 10 0.6625 -n 11 0.625 -n 12 0.5875 -n 13 0.55 -n 14 0.5125 -n 15 0.475 -n 16 0.4375 -n 17 0.4 -n 18 0.3625 -n 19 0.325 -n 20 0.2875 -n 21 0.25 -n 22 0.2125 -n 23 0.175 -n 24 0.1375 -n 25 0.1 -ej 6.25E-5 25 24 -ej 1.25E-4 24 23 -ej 1.875E-4 23 22 -ej 2.5E-4 22 21 -ej 3.125E-4 21 20 -ej 3.75E-4 20 19 -ej 4.375E-4 19 18 -ej 5.0E-4 18 17 -ej 5.625E-4 17 16 -ej 6.25E-4 16 15 -ej 6.875E-4 15 14 -ej 7.5E-4 14 13 -ej 8.125E-4 13 12 -ej 8.75E-4 12 11 -ej 9.375E-4 11 10 -ej 0.001 10 9 -ej 0.0010625 9 8 -ej 0.001125 8 7 -ej 0.0011875 7 6 -ej 0.00125 6 5 -ej 0.0013125 5 4 -ej 0.001375 4 3 -ej 0.0014375 3 2 -ej 0.0015 2 1*

**Two-Dimensional population grid (Model two)**

For a fixed grid of five by five populations (see Figure 2B), we established bidirectional migrations between neighbor populations at *m* migration rates, and run the ms software using a stepping stone model. Ne was set to 2,000 chromosomes for each population, and only *m* was changed from0.005 to 0.025 to allow increasing migration rate between neighbor populations. Since we are interested in the scaled migration rate by the effective population size, either of the two parameters could have been changed from simulation to simulation.

For the case *m* = 0.005, the ms command is:

*ms 500 10000 -t 10.0 -I 25 20 20 20 20 20 20 20 20 20 20 20 20 20 20 20 20 20 20 20 20 20 20 20 20 20 -m 1 2 40.0 -m 2 1 40.0 -m 1 6 40.0 -m 6 1 40.0 -m 2 3 40.0 -m 3 2 40.0 -m 2 7 40.0 -m 7 2 40.0 -m 3 4 40.0 -m 4 3 40.0 -m 3 8 40.0 -m 8 3 40.0 -m 4 5 40.0 -m 5 4 40.0 -m 4 9 40.0 -m 9 4 40.0 -m 5 10 40.0 -m 10 5 40.0 -m 6 7 40.0 -m 7 6 40.0 -m 6 11 40.0 -m 11 6 40.0 -m 7 8 40.0 -m 8 7 40.0 -m 7 12 40.0 -m 12 7 40.0 -m 8 9 40.0 -m 9 8 40.0 -m 8 13 40.0 -m 13 8 40.0 -m 9 10 40.0 -m 10 9 40.0 -m 9 14 40.0 -m 14 9 40.0 -m 10 15 40.0 -m 15 10 40.0 -m 11 12 40.0 -m 12 11 40.0 -m 11 16 40.0 -m 16 11 40.0 -m 12 13 40.0 -m 13 12 40.0 -m 12 17 40.0 -m 17 12 40.0 -m 13 14 40.0 -m 14 13 40.0 -m 13 18 40.0 -m 18 13 40.0 -m 14 15 40.0 -m 15 14 40.0 -m 14 19 40.0 -m 19 14 40.0 -m 15 20 40.0 -m 20 15 40.0 -m 16 17 40.0 -m 17 16 40.0 -m 16 21 40.0 -m 21 16 40.0 -m 17 18 40.0 -m 18 17 40.0 -m 17 22 40.0 -m 22 17 40.0 -m 18 19 40.0 -m 19 18 40.0 -m 18 23 40.0 -m 23 18 40.0 -m 19 20 40.0 -m 20 19 40.0 -m 19 24 40.0 -m 24 19 40.0 -m 20 25 40.0 -m 25 20 40.0 -m 21 22 40.0 -m 22 21 40.0 -m 22 23 40.0 -m 23 22 40.0 -m 23 24 40.0 -m 24 23 40.0 -m 24 25 40.0 -m 25 24 40.0*
